# Experts’ consensus on the definition and management of high risk multiple myeloma

**DOI:** 10.3389/fonc.2022.1096852

**Published:** 2023-01-23

**Authors:** Chiara Marcon, Valentina Simeon, Paola Deias, Gabriele Facchin, Alessandro Corso, Daniele Derudas, Vittorio Montefusco, Massimo Offidani, Maria Teresa Petrucci, Renato Zambello, Raffaella Stocchi, Renato Fanin, Francesca Patriarca

**Affiliations:** ^1^ Division of Hematology, S. Maria della Misericordia Hospital, Azienda Sanitaria Universitaria Friuli Centrale, Udine, Italy; ^2^ Department of Area Medica, Udine University, Udine, Italy; ^3^ Division of Hematology and Bone Marrow Transplant Center, Department of Medical Science, R. Binaghi Hospital, Cagliari University, Cagliari, Italy; ^4^ Division of Hematology, Legnano’s Hospital, Milan, Italy; ^5^ Division of Hematology and Bone Marrow Transplant Center, A. Businco Cancer Hospital, Cagliari, Italy; ^6^ Division of Hematology, Azienda Socio Sanitaria Territoriale (ASST) Santi Paolo e Carlo, Milan, Italy; ^7^ Clinical Hematology, Azienda Ospedaliera Universitaria (AOU) Ospedali Riuniti di Ancona, Ancona, Italy; ^8^ Division of Hematology, Department of Translational and Precision Medicine, Azienda Ospedaliera Policlinico Umberto I, Sapienza University of Rome, Rome, Italy; ^9^ Clinical Hematology, Azienda Ospedaliera di Padova, Padua, Italy

**Keywords:** high risk multiple myeloma, experts’ consensus, R-ISS staging, Delphy method, double hit multiple myeloma

## Abstract

High risk multiple myeloma (HRMM) at diagnosis is currently recognized according to the Revised International Staging System (R-ISS) which was set up in 2015. Since then, new clinical and biological prognostic factors have been developed, which could implement the definition of High Risk (HR) category. We conducted a survey in order to identify which additional parameters, both clinical and biological, are considered more useful for the clinical practice and to evaluate if the management of Multiple Myeloma (MM) should change on the basis of the risk category. A questionnaire, consisting of 8 statements, was submitted to 6 Italian experts, from the European Myeloma Network (EMN) Research Italy, using the Delphi method. The colleagues were asked to answer each question using a scale between 0 and 100. If a statement did not reach at least 75 out of 100 points from all the participants, it was rephrased on the basis of the proposal of the experts and resubmitted in a second or further round, until a consensus was reached among all. From the first round of the survey a strong consensus was reached regarding the opportunity to revise the R-ISS including chromosome 1 abnormality, TP53 mutation or deletion, circulating plasma cells by next generation flow and extramedullary plasmacytomas. No consensus was reached for the definition of “double hit” MM and for the application in clinical practice of treatment strategies based on the risk category. In the second round of the Delphi questionnaire, “double-hit” MM was recognized by the association of at least two high-risk cytogenetic or molecular abnormalities. Moreover, the experts agreed to reserve an intensified treatment only to specific conditions, such as plasma cell leukaemia or patients with multiple extramedullary plasmacytomas, while they admitted that there are not sufficient real word data in order to modify treatment on the basis of MRD assessment in clinical practice. This survey suggests that the definition of HRMM should be implemented by additional clinical and biological risk factors, that will be useful to guide treatment in the future.

## Introduction

Multiple Myeloma (MM) is a plasma cell dyscrasia, characterized by a variable clinical course alternating periods of remission due to treatments and disease progressions. MM is still considered an incurable disease, since free intervals are likely to become shorter and shorter because of drug resistance occurrence up to the patient’s death. In 2022, MM accounts for approximately 1.8% of all new cancer cases and 2.1% of all cancer deaths. Relative survival at 5 years, calculated between 2012–2018, is 57.9%^1^, with a few patients alive more than 20 years after diagnosis and most cases surviving with relapsed or refractory disease. This heterogeneous clinical course is linked to MM biology and can be predicted at diagnosis by some prognostic factors.

High risk multiple myeloma (HRMM) at diagnosis is currently recognized according to the Revised International Staging System (R-ISS) which was set up in 2015 (1). Since then, new clinical and biological prognostic factors have been developed, which could modify the definition of HR category.

In recent years, thanks to the development and diffusion of innovative diagnostic tools, new features of disease at onset have been studied. Some of these have proven to be prognostically unfavourable, such as: some karyotypic and genetic alterations not included in R-ISS, circulating plasma cells and presence of extramedullary disease. Moreover, the definition of complete response after treatments can be improved by the study of minimal residual disease (MRD) by immune phenotypic or molecular techniques, and MRD persistence has been invariably linked to shorter relapse times, accounting for a dynamic criterion of high risk.

Thanks to these new parameters, the definition of the HRMM has become more and more complex, leaving several questions still open.

We conducted a survey using the Delphi method by submitting a questionnaire to 6 Italian experts, belonging to the European Myeloma Network (EMN) in order to identify which additional clinical and biological parameters have been considered more useful for the clinical practice and to evaluate if the management of MM has been changed on the basis of the risk category.

## Methods

The study was articulated in 3 phases. First, a Pubmed literature review on HRMM was conducted, including original articles, reviews and guidelines published in English. Eight issues were identified. Four of which were represented by the following unfavourable prognostic factors currently not included in the main prognostic risk scores: chromosome 1 alterations, TP53 mutations, circulating plasma cells and presence of extramedullary disease. Four further areas of interest were individualised: limits of R-ISS, double-hit MM definition, role of therapeutic strategies differentiated on the basis of risk at diagnosis or on MRD results after treatments.

**Figure 1:**
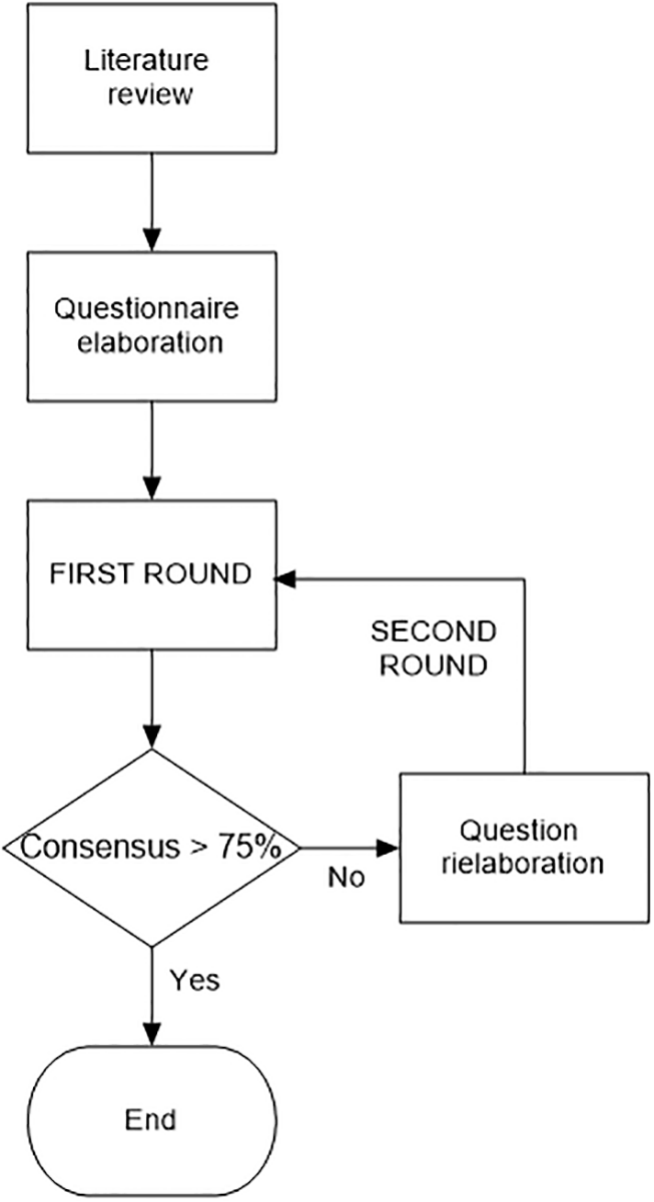
Delphi method run.

Second, we made a statement for each issue and looked for consensus among 6 Italian MM experts using the Delphi technique. The Delphi technique (2, 3) is a structured method to define a consensus based on group opinion by surveying a panel of experts. It’s based on subsequent questionnaires in a series of rounds: the responses to one round are used to produce the questionnaire for the next round, based on the specialists’ feedback. (**Figure 1**)

**Figure 2:**
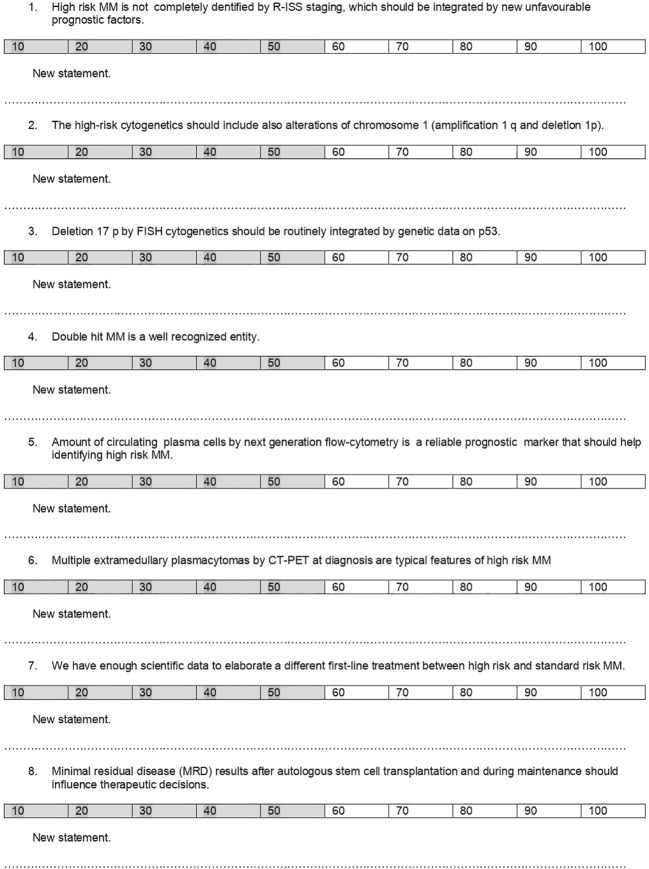
Delphi questionnaire.

Third, the questionnaire (**Figure 2**) was emailed to 6 colleagues belonging to the European Myeloma Network (EMN) and working in the northern and central part of Italy. Affiliation to the same research group and operating in a homogeneous geographical area allows adherence of experts to the common guidelines of clinical practice.

The 6 colleagues were asked to score each question using a scale between 0 and 100. If a statement did not reach at least 75 out of 100 points from all the participants, it was rephrased on the basis of the proposal of the experts and resubmitted in a second or further round of the Delphi questionnaire until a consensus was reached among all. Consensus on each topic was reached if the average score among the 6 experts was ≥ 75%.

## Results

### Beyond the conventional staging systems

Background:

Finding a complete staging system able to define high-risk MM doesn’t seem to be that simple. Being a heterogeneous disease, there are several elements that must be considered. Over time, various risk stratification systems have followed one another. After the historical Durie-Salmon staging (which included protein M levels, number of lytic bone lesions, haemoglobin values, serum calcium levels, and creatinine) the International Staging System (ISS), based on levels of albumin and Beta2 microglobulin, was introduced in 2003.

Moreover, following the evidence of the great prognostic impact of some chromosomal abnormalities on the course of the disease – identified thanks to the rapid development of fluorescence *in situ* hybridization (FISH) karyotyping – this score was implemented by the International Myeloma Working Group (IMWG) to the Revised-ISS (R-ISS). So, the following high risk cytogenetic abnormalities have been added to the previous ISS system: translocation (4;14), translocation (14;16) and deletion 17p (del17p). In addition, high levels of serum lactate dehydrogenase was introduced as a factor, since it was associated with shorter survival and high proliferation rate, more aggressive tumour and extramedullary disease (4).

The risk stratification is even more important since the huge advance in the drugs armamentarium allows to hypothesize a differentiated therapeutic approach according to the risk.

However, the R-ISS has shown some limitations. Several groups of experts, like those of the Intergroupe Francophone du Myelome, have underlined the opportunity to add new prognostic factors, even if a consensus has not been reached or published yet (5). In fact, other characteristics, some peculiar to the tumour, others to the patient himself, which can influence the course of the disease, had not been considered yet.

Among disease characteristics, there are other FISH chromosomal abnormalities (CA) conferring worse survival, such as deletion 1p, amplification 1q and the rare translocation (14;20). Furthermore, deletion 17p seems to have a different prognostic impact according to the percentage of marrow plasma cells carrying the abnormality and if the FISH alteration is associated with a mono or biallelic inactivation of p53, identified thanks to the advent of Next Generation Sequencing (NGS). In the same way, quantity may change the prognostic significance of 1q+, according to the number of alterations involved.

Recently, R-ISS score was implemented, developing R2-ISS (6). In this new prognostic score the following significant predictors for OS and PFS are considered: ISS, LDH, del(17p), t(4;14) and 1q+, while the evaluation of t(14; 16) is escluded, having a minor impact on PFS. Considering these parameters, new diagnosed Multiple Myeloma (NDMM) are divided into low risk (R2-ISS I, 0 points), intermediate-low risk (R2-ISS II, 0.5-1 points), intermediate-high risk (R2-ISS III, 1.5 -2.5 points) and high risk (R2-ISS IV, 3-5 points).

In addition, with the introduction of gene expression profiling (GEP) applied on myeloma plasma cells, several research groups (HOVON, UAMS and others) were able to detect genes frequently involved in MM pathogenesis and to better identify HR patients. However, these signatures are not always overlapping, therefore, they remain a research technology tools, that is still being implemented before entering the clinical practice (7).

**Figure 3:**
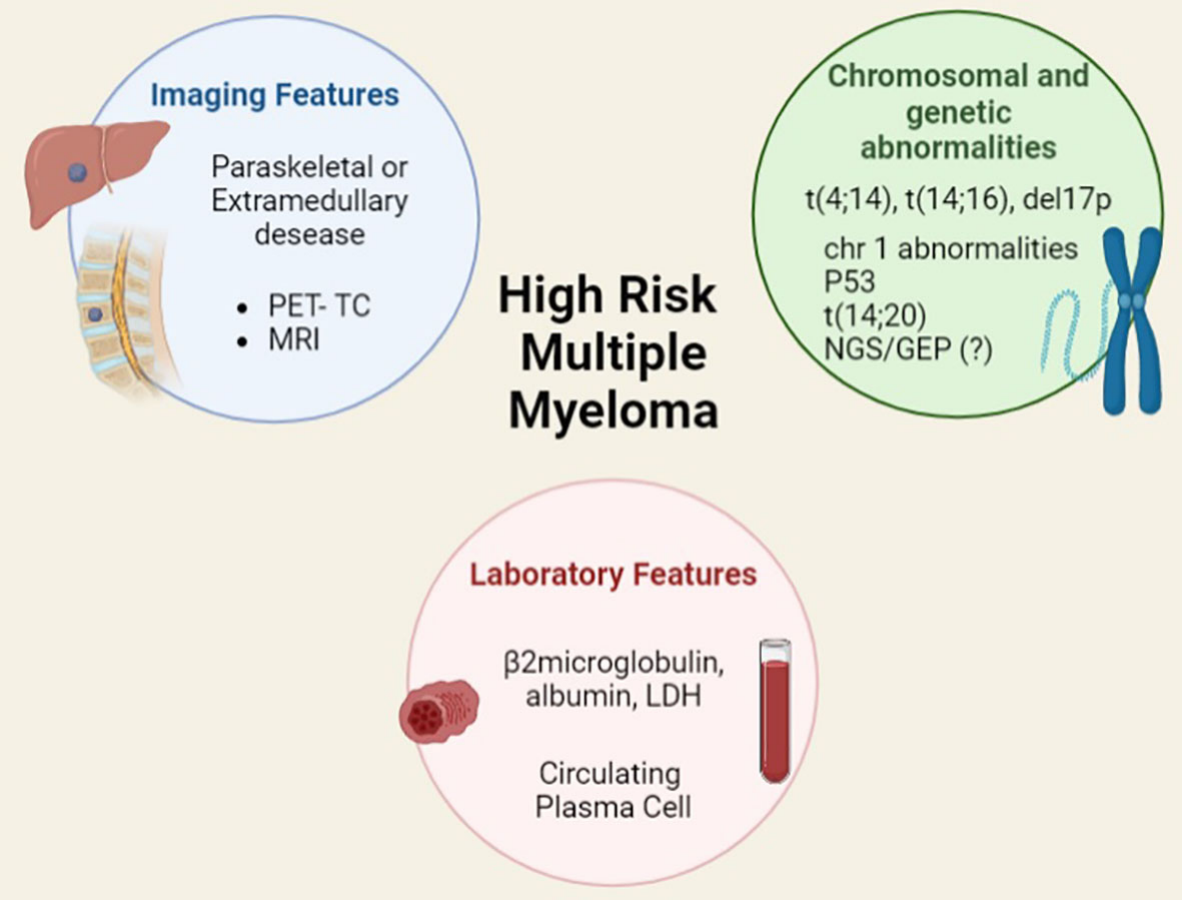
Features, included and not included in current prognostic scores, for identifying high risk multiple myeloma.

Moreover, MM clinical features such as the extramedullary involvement either of soft tissues or viscera identified by PET-CT and MRI scans have demonstrated a prognostic significance at diagnosis and at time of relapse. Plasma cell (PC) leukaemia, conventionally defined as at least 2000 circulating PC/microliter and/or 20% or more PC is a well-known very aggressive phenotype; however, the new and more sensitive techniques of flow cytometry (FC), such as the next generation FC, have allowed to show that even a lower rate of circulating PC has a strong and independent impact on the outcome. (8, 9) Other elements that deserve to be studied are PC immunophenotype, microvessels density, type of monoclonal protein, and serum free light chain ratio (**Figure 3**).

Among the patient factors, chronological age seems to be surpassed by biological age, defined on the basis of the coexistence of comorbidities and organ function impairment, and measured by different frailties scores, such as those proposed by the IMWG and the German group (10, 11).

However, these scores are not always easy to apply, because of variable judgment among different observers, need of time by the physicians, and influence by disease burden, so they should be standardized and applied in prospective studies (12).

A further aspect that must be considered is that the definition of risk is not only established at the time of diagnosis but it must be redefined in the course of disease in a dynamic way, including the response to therapy, integrated by imaging findings and MRD (7).

Accepted recommendation: High risk MM is not comprehensively identified by R-ISS staging, which should be integrated by new unfavourable prognostic factors.

Round of Consensus: 1

Grade of Consensus: 96.66%

### Chromosome 1 alterations

Background:

Among the CAs that define the prognosis of MM, the alterations of chromosome 1 are not currently included in R-ISS. These CAs, and the related genetic anomalies, play a major role in the onset and course of the disease. In fact several genes such as CKS1B, PSMD4, IL6R, MCL1 involved in proliferation and survival of tumour cells, mechanisms of chemo refractoriness, and alteration of the microenvironment are located in the 1q21 region (13). Moreover, more than 30% of the 70 genes studied by HOVON65/GMMG-HD trial GEP were mapped on chromosome 1 (14). The acquisition or amplification of chromosome 1q21 is also one of the most frequent CAs affecting about 40% of patients with a NDMM and it can be found in most cases of smouldering MM before they evolve into symptomatic MM. The occurrence of this CA increases with the evolution of the disease, affecting over 70% of relapsing patients (15).

The prognostic significance of 1q amplification is probably related to a “dosage effect” of these genes, in particular CKS1B, since it has been shown that the presence of ≥3 copies of 1q is linked to a worse outcome than the gain of 1 to 3 copies (16, 17). Moreover, the gain of chromosome 1q21 makes that gene region more unstable, resulting in the accumulation of additional copies of 1q21 as the disease progresses, leading to a true amplification of the chromosome region (> 3 copies of chromosome 1q21). Therefore, the accumulation of chromosome 1q amplicons is indicative of a state of major chromosomal instability and high mitotic index. In recent years, the importance of chromosome 1q21 alteration as an unfavourable prognostic factor has been investigated; several evidences indicate 1q21 amplification (four or more copies) as a poor prognostic factor defining high-risk disease (18, 19), while evidences are less strong for unfavourable prognostic significance of 1q21 gain (1 to 3 copies) (20). The alterations of 1q (considered overall as “Gain 1q”) are already included in the prognostic scores of the Mayo Clinic mSMART 3.0^2^, the French group (5) and the European Myeloma Network (6). The inclusion of amp1q is one of predictors of the R2-ISS score, that has recently allowed a better NDMM stratification. Nevertheless, in R2-ISS definition it is not currently specified if the number of 1q copies involved should have a different prognostic impact (6).

Presence of amp1q seems to have therapy implications, suggesting the effectiveness of an intensification with early Autologous Stem Cell Transplant (ASCT) and subsequent Bortezomib as consolidation or maintenance (21). At the moment, as no prospective trials are available, there is no certainty of being able to overcome the negative prognostic impact of this mutation with a therapy with proteosome inhibitors (although some studies suggest it (22)).

The deletions of the p arm (del1p12, del1p21, del1p22, del1p31, 1p32) are included among the alterations of chromosome 1 and affect 5% of MM patients (23). These mutations can be acquired (with or without association) with amp1q. The del1p (all break-points) correlates independently with a poor prognosis (24). Despite their strong prognostic value, del1p is not currently included in the mSMART and R2-ISS prognostic scores, while the more frequent 1p32^6^ is included in the French score.

In conclusion, alterations of chromosome 1 are prognostically unfavourable, due to aggressive clinical presentation and poor response to therapy.

Accepted recommendation: The high-risk cytogenetics should also include alterations of chromosome 1 (amplification 1 q an d deletion 1p).

Round of Consensus: 1

Grade of Consensus: 96.66%

### Deletion 17p

Background:

Deletion of 17p chromosome (del17p), a recurrent cytogenetic abnormality present in about 10% of patients at diagnosis and in up to 80% of patients with relapsed and/or refractory disease and in secondary plasma cell leukaemia, is an unfavourable prognostic factor recognized by the R-ISS (4, 25).

Patients with del17p at diagnosis usually show advanced MM stages and aggressive clinical features and present the shortest progression-free survival and overall survival among all the groups with other cytogenic abnormalities (25).

However, the clonal size carrying del17p and its prognostic impact are still a matter of debate. In fact, even if most studies recognized that a percentage of plasma cells with del17p greater than 20% confers a bad prognosis, other researchers have suggested to set the threshold at 55%-60% (12, 25, 26). In order to define the prognostic impact of del17p, it is useful to consider the alterations of the involved genes, that can be studied by NGS. The TP53 gene, which encodes for the p53 protein, is found on chromosome 17p(13.1). P53 is a protein that acts as a tumour suppressor, safeguarding the integrity of the genome.

Its signalling pathway is inactivated during physiological conditions thanks to several control mechanisms. Once activated, in response to oncogenic stress or DNA damage, p53 induces several tumour suppressor actions, like cell-cycle arrest, apoptosis, senescence and inhibition of angiogenesis, contributing to prevent tumour development and maintaining cellular homeostasis (25, 26).

Germinal mutations of TP53 have been shown to be closely related to the development of cancers, since it is mutated approximately in half of human tumours (26).

Even if TP53 mutations and deletions are less common in haematological neoplasms as compared to solid neoplasms, they can be found in Burkitt’s lymphoma, chronic myeloid leukaemia, adult T-cell leukaemia, B-cell prolymphocytic leukaemia, and chronic lymphocytic leukemia (26). In MM p53 may play an important role in disease pathogenesis and evolution (26). In fact, TP53 alterations are rarely detectable at diagnosis but their incidence increases in late stages, highlighting its critical role in disease progression. Biallelic inactivation of TP53 at diagnosis, expressed with loss plus mutation of TP53 (“double hit”), represents an important marker of adverse prognosis when compared to wild-type or monoallelic inactivation. Again, mutations are often missense mutation with gain of function, making this gene an oncogene. In a lower percentage, some mutations are nonsense mutations, making p53 truncated proteins (27).

According to some data, TP53 mutation seems to occur concomitantly or subsequently to the deletion of 17p13 (28–30). Patients with biallelic TP53 inactivation have a poor outcome with median overall survival of 36 months, compared to the median OS of 53 months of patients with del 17p alone (7).

Accepted recommendation: Deletion 17 p by FISH cytogenetics should be routinely integrated by the evaluation of mutation and/or deletion of TP53.

Round of Consensus: 1

Grade of Consensus: 91.66%

### Definition of double-hit multiple myeloma

Background:

The “double-hit” terminology has been used to define a specific category of non Hodgkin lymphomas with a very poor prognosis, due to the simultaneous presence of two genetic mutations. There is also a further subgroup with the simultaneous presence of 3 genes with poor prognosis, defined as “triple hit” lymphomas. Specific and intensified therapeutic strategies have been developed to face the clinical and biological aggressiveness.

Similarly to lymphomas, in 2018, Walker et al. (31) defined the “double-hit” and “triple-hit” subgroup of high-risk MM, including those patients who simultaneously presented two or more prognostically unfavourable CAs and had a very aggressive course with median OS of 15.4 months. Compared to high-risk MM, the “double-hit” and “triple-hit” categories identify a small group of patients of less than 10% with an extremely poor prognosis. For this reason, these definitions have been included in the most recent prognostic classification mSMART.

However, the concept of “double-hit” MM is not always uniformly accepted in literature. For some authors, “double-hit” definition overlaps with the of “ultra/very high risk” MM, that includes not only the presence of more than one CA abnormalities but also the presence of one CA alteration associated with ISS 3 or clinical aggressive presentation (5). For other authors, “double-hit” includes the gene alterations identified by NGS, such as del17p/TP53 and amp1q (32, 33), or molecular alteration such as mutation in CRBN and Ras revelated by GEP (30)

Similarly to lymphomas, “double-hit” MM will probably benefit from a specific and intensified treatment either at induction (including early ASCT) or maintenance (34, 35); although, right now no prospective trials are available.

Rejected recommendation: Double hit MM is a well recognized entity.

Round of Consensus: 1

Grade of Consensus: 55%

Accepted recommendation: Double hit MM is identified by the presence of at least 2 high risk cytogenetic or molecular abnormalities.

Round of Consensus: 2

Grade of Consensus: 96.66%

### The prognostic role of circulating plasma cells

Background:

There is increasing evidence of the prognostic importance of circulating plasma cells (CPC). The conventional diagnostic threshold of 20% peripheral PC necessary for the definition of Plasmacell Leukemia (PCL) has been recently revised and, currently, presence of 5% or more CPC is sufficient for the diagnosis (8). Similarly, in patients with MM, the presence of CPC is linked to shorter OS, such as in PCL, as demonstrated by both a Spanish (36) study and a Mayo Clinic (37) study, that independently defined a significant cut-off of 5% CPC.

The biological role of CPC in disease progression is not completely understood, but it probably reflects the acquired independence from marrow microenvironment and the tumour burden. The presence of CPC has been demonstrated as an unfavourable prognostic factor in plasma cell disorders, including MGUS, smouldering or active MM (9). Many retrospective data have consistently shown that, in newly diagnosed MM, detectable CPCs at diagnosis have a negative prognostic impact on PFS and OS, supporting a role as a biomarker in baseline risk-evaluation (38).

On this basis, the attempt of integrating the CPC levels to current prognostic systems was made in order to allow a better risk stratification; a relevant improvement in predictive power was found in intermediate risk-class, i.e. R-ISS II or standard cytogenetic risk, identifying patients with a worse outcome (39–41).

Peripheral blood involvement by tumoral plasma cells appears to be underreported using morphological cytology on blood smear, while CPC detection seems to reach up to 40% in newly diagnosed MM samples using highly sensitive methods (42), that could also discriminate polyclonal PCs. The quantification of circulating plasma cells at diagnosis with more sensitive techniques such as multiparameter (MPF cytometry) or Next-generation flow cytometry (NGF) could detect the presence of CPCs with a cut-off of 10^-5^-10^-6^, providing a tool for a better detection of this high-risk feature. However, a clear threshold could not be identified and probably the CPC level could be better considered as a continuous variable (43).

A recent analysis of patients enrolled in FORTE trial showed that the evaluation of baseline CPCs with MPF could support risk stratification, identifying a cut-off of 0.07% (approximately 5 cells/uL) CPC predicting a worse PFS. Consistently with these results, CPCs detection by NGF in the context of Spanish GEM2012MENOS65 and GEM2014MAIN trials found that a cut-off of 0.01% was predictive of shorter PFS. In both trials, the adverse impact of baseline detectable CPCs was reduced only by the achievement of MRD negativity, suggesting the need for treatment intensification on these patients (44).

Given these findings, the quantification of CPCs emerges as a new biomarker of aggressive disease, even if it requires methodological standardization and validation in the context of prospective clinical trials.

Accepted recommendation: Amount of circulating plasma cells by next generation flow-cytometry is a reliable prognostic marker that should help identify high risk MM.

Round of Consensus: 1

Grade of Consensus: 75%

### Extramedullary disease

Background:

Extramedullary Disease (EMD) has a well-recognized unfavourable prognostic significance independently from any other adverse risk factor, both at diagnosis and in relapsed refractory phase (45). In many studies, EMD was associated with a shorter OS and PFS, with an inferior outcome observed even in standard-risk patients in comparison to patients without EMD, despite the availability of therapeutic combinations with proven clinical efficacy, such as reported in Total Therapy 3 protocols and, more recently, with novel agents-based induction (46–48). A significant advantage was observed with double ASCT intensification, probably according to high-risk cytogenetic features reported in almost 40% of EMD patients that can benefit from tandem over single procedure (49). Furthermore, the presence of EMD before allogeneic SCT was significantly associated with an unfavourable outcome, with median OS inferior to 8 months (50).

A consensus has been recently reached to overcome different definitions of EMD, distinguishing paraskeletal disease or bone-related plasmacytomas, growing contiguous to bone focal lesion, and soft-tissue plasmacytomas originating from hematogenous spread of malignant plasma cells (51, 52). The hematogenous spread can involve skin, subcutaneous tissue, liver, breast, kidney, pleura, lympho nodes or central nervous system (CNS) 51. Rather than being only an anatomical description, this distinction can also have a clinical impact on outcome, as demonstrated by several independent studies.

In a large meta-analysis of 8 GIMEMA and EMN trials, EMD “per se” did not appear to impair global PFS with the use of novel agents as a part of therapeutic strategy, and even more when followed by intensification with ASCT. However, these apparently conflicting results can be explained by the high rate of bone-related extramedullary lesions reported in this population, and, due to low use of PET/CT in older trials, the underestimation of extramedullary plasmacytomas, which retained a worse impact on OS when evaluated independently in multivariate analysis (53).The prognosis is particularly poor in patients with CNS involvement who have a median survival of <3 months even after treatment with novel agents 51.

Similar results derived from an EBMT Registry analysis including 682 transplant-eligible patients with EMD at diagnosis. In this study, patients with single paraskeletal localization seemed to have a similar 3y-PFS in comparison to patients without EMD, in contrast to multifocal paraskeletal localizations and single soft tissue plasmacytoma that are associated with a negative impact on both 3y PFS and OS. These findings suggest that intensification with ASCT can have a beneficial impact in this setting of high-risk patients and that the use of radiation in single site localization might impact response and therefore contribute to better PFS (54). More recently, a multicentre real world retrospective survey clearly showed a different prognosis for paraosseous and soft tissue plasmacytomas, with a reported benefit in transplant-eligible patients from receiving a double procedure, consistent with EBMT data (55).

The importance of PET/CT in the evaluation of patients with multiple myeloma relies on its ability to define tumour burden detecting bone lesions and EMD with high sensitivity, and at the same time to characterize disease activity with metabolic parameters, with regard to size, number, and metabolic uptake (SUV) of each lesion (56).

On this basis, the role of PET/CT in defining clinical response and as a marker of MRD is strongly assessed with the clear recognition of the unfavourable impact on PFS and OS of PET/CT positivity at different time-points during disease course (after induction, after ASCT or after consolidation) complementarily with bone marrow MRD detection (57). PET/CT positivity retains its poor prognostic impact even in the setting of allogeneic stem cell transplantation, in particular considering the ability of EMD detection and metabolic disease activity expressed by SUVmax (58).

PET/CT parameters could integrate conventional risk factors to identify high-risk patients at diagnosis better than other imaging techniques.

A first comparison between MRI and PET/CT imaging at diagnosis in a large prospective trial suggested that the presence of more than 7 MRI detected focal lesions or more than 3 FDG-avid focal lesions correlated with a shorter PFS (59).

An Italian prospective study comprising transplant-eligible patients in the context of novel agent-based induction further supported the prognostic significance of PET/CT scan, showing a significant association between >3 focal lesions, >4.2 SUV max and presence of EMD with inferior OS and PFS, with a confirmed role for EMD and metabolic avidity also in multivariate analysis (60). Similar results on the prognostic value of SUVmax in NDMM patients derived from a combined harmonized analysis of two prospective phase III trials (IFM/DFCI2009 and EMN02/HO95) (61).

The French IMAJEM study, which considered both MRI or PET/CT for bone lesions detection, confirmed that the presence of EMD detected at diagnosis with PET/CT was independently related with shorter PFS and OS, while PET/CT (but not MRI) normalization after induction and before maintenance, was associated with better PFS (62).

More recent results from CASSIOPET prospective substudy of the CASSIOPEIA trial demonstrated that negative baseline PET translated into a better PFS even with the use of a very effective quadruplet induction, including an antiCD38 monoclonal antibody in first line treatment (63).

In order to harmonize imaging reporting in clinical practice, new criteria for PET/CT interpretation and standardization have recently been proposed by the Italian group (Italian myeloma criteria for PET use or IMPeTUs), including a visual interpretation according to Deauville 5-point System scoring, FDG distribution description as bone marrow non focal uptake, focal bone lesions, and distinction between paramedullary and extramedullary lesions. Deuville criteria were validated using a large population enrolled in phase III IFM/DFCI2009 and EMN02/HO95 trials and proved to be reproducible with high interobserver concordance (64).

In conclusion, available data on clinical outcome of EMD derived mostly from retrospective studies or from subgroup analysis; results are therefore biased by small population size, heterogeneity of disease definition, and treatment options considered, with a scarcity of studies directly powered to address this subset of patients. However, the strong evidence of the unfavourable impact of multiple lesions with particular regard to true extramedullary ones, and the potential of FDG-PET/CT to combine anatomical detection with functional and molecular data should suggest its incorporation into prognostic evaluation in newly diagnosed myeloma patients.

Accepted recommendation: Multiple extramedullary plasmacytomas by CT/PET at diagnosis are typical features of high-risk MM

Round of Consensus: 1

Grade of Consensus: 95%

### Treatment strategy based on MM risk at diagnosis

Background:

After the publication of the clinical results of the CASSIOPEIA study (65) (comparison Dara-VTD *vs* VTD) and the GRIFFIN trial (66) (Dara-VRD *vs* VRD), the recommended induction treatment for transplant-eligible patients has become the quadruplet-drug therapy including VTD and Daratumumab. Moreover, in the European guidelines (67), the recommended first-line treatment for non-transplant-eligible patients is Daratumumab with RD according to the MAIA trial (68), or Daratumumab with VMP according to the ALCYONE trial (69). Guidelines clearly indicate the need to adjust dosage or save some of the drugs according to the clinical status of the patients (age, frailty, comorbidities, or MM related complications) but the recommended induction does not change based on the clinical or cytogenetic risk.

In the CASSIOPEIA trial (65), high-risk subgroups presenting unfavourable CAs or ISS 3 were analysed: the use of Dara-VTD demonstrates advantage in terms of both PFS and MRD negativity, suggesting a probable benefit due to the intensification of treatment for HRMM. Moreover, new quadruplets are being studied, such as the EMN24 study^3^ comparing Isatuximab-KRd *vs* KRd induction autologous transplant in both arms followed by Isatuximab-KRd *vs* KRd maintenance at reduced doses. Indeed, a sequential treatment Isatuxima-KRD-based has been experimenting in a phase 2 study by the German group in patients with ISS 2 or 3 stage in association with deletion 17, translocation 14 or amplification 1 q. The clinical results of the interim analysis on the first 50 patients were reported showing 50% CR and 64% MRD negativity after induction consisting of 4 Isatuximab-KRD cycles (reference). These preliminary results are promising and underline the importance of designing trials specifically dedicated to high risk patients (70).

The European guidelines suggested to intensify the treatment in HRMM patients performing a double autologous transplant according to the results of the EMN02/HO95 study (71) and the BMT-CTN 0702 trial (72), demonstrating advantage in terms of PFS and OS in the subset of patients with high risk cytogenetic. However, since the studies did not include monoclonal antibodies, we currently do not know if these results can be translated to the new strategies adding Daratumumab or Isatuximab.

Another possibility to intensify the treatment in HRMM is a more aggressive maintenance, substituting lenalidomide or combining other drugs to it.

A few evidences suggested that proteasome inhibitors may overcome the unfavourable prognosis linked to CA. In fact, the long term clinical results of the HOVON-65/GMMG-HD4 study (73) showed that bortezomib induction, double ASCT, and bortezomib maintenance for 2 years significantly improved PFS and OS in patients with del(17p) and creatinine higher 2 mg/dL, but not in patients presenting with t(4;14) and add(1q).

Maintenance with other proteasome inhibitors, such as the oral Ixazomib, achieved promising results in randomized studies in comparison to observation both in transplant-eligible patients and in the HR-subgroup. Maintenance therapy with Ixazomib *vs* Ixazomib plus Daratumumab has been recently compared in the EMN18 trial^4^, but results are not mature yet.

Patients with multiple sites of EMD or those with circulating PC at diagnosis may receive a different first-line treatment approach. These HRMMs (both EMD and PCL) are characterized by poor response to conventional therapy and/or early relapses, therefore some experts suggest a debulking with chemotherapy such as PACE associated with monoclonal antibodies, followed by a consolidation with autologous transplantation (74). Interestingly, tandem-ASCT appears to be more effective in terms of PFS (49) in EMD MM, in contrast with previous evidence (54).

Allogeneic transplantation (ALLO) has been experimented in patients with HRMM, although evidences of superiority over ASCT are conflicting. Among the 5 prospective studies comparing tandem ASCT versus ASCT followed by ALLO at diagnosis, only 2 trials (75, 76) showed prolonged PFS and OS in the ALLO arm. However, patients were heterogeneous and high-risk MM was not evaluated according to current staging and cytogenetics, since these large trials were conducted during the first decade of the twentieth century. In the long-term follow-up of the German study (77), a better outcome was observed in patients harbouring del 17 p and treated with auto/allo, even if the sample was quite small (19 patients). Moreover, no advantage was observed in plasma cell leukemias treated with ALLO over ASCT in the CIMTR registry (78).

In the near future, another therapy could be a weapon for MM eradication in HR MM: CAR-T cells. In Europe, CAR-T cells against BCMA antigen have been tested in the KarMMa and Cartitude trials in triple-class relapsed/refractory MM, showing promising results also in patients with high-risk cytogenetics (79, 80), that should be confirmed by real world data in larger groups of patients.

Rejected recommendation: We have enough scientific data to elaborate a different first-line treatment between high risk and standard risk MM.

Round of Consensus: 1

Grade of Consensus: 46.66%

Accepted recommendation: At present we do not have enough scientific data to elaborate a different first-line treatment between high-risk and standard-risk MM in all patients; however, a few very high-risk conditions, such as plasma cell leukaemia or multiple extramedullary plasmacytomas at diagnosis, should receive an intensified treatment.

Round of Consensus: 2

Grade of Consensus: 93.33%

### Treatment strategy based on minimal residual disease evaluation

Background:

The assessment of response to therapy in MM is complex. Response criteria were last revised in 2016 by the IMWG. Complete response (CR) was defined as the contemporary presence of negative immunofixation (both serum and urinary), <5% plasma cells in bone marrow aspirate and disappearance of any soft tissue plasmacytoma (81). A new category of deeper remission named stringent CR was introduced, characterized by CR criteria, normal free light chain ratio, and absence of clonal PC in BM by immunochemistry or immunofluorescence (81).

However, these criteria have several limits. Firstly, the serum monoclonal component is an indirect measure of the persistence of secreting plasma cells after treatment and serum immunofixation can be falsely positive (82), especially after monoclonal antibodies (83). Furthermore, in recent years, the role of urinary immunofixation in defining the complete response has been discussed (84). Secondly, morphological evaluation of bone marrow aspirate is not sufficiently sensitive in defining the amount of residual plasma cells after therapy, as occurs in other haematological malignancies. Finally, MM is localized not only in the bone marrow disease but also in bones and extramedullary sites that should be restaged after therapy as well as bone marrow (60, 85).

For these reasons, the evaluation of MRD was introduced in the response criteria developed by IMWG (81).

MRD negativity, defined with NGS or NGF, indicates a deeper response than standard CR, with a sensitivity between 10^-5^ and 10^-6^. The results of NGS and NGF are comparable, although NGS is technically more applicable and can be performed also in frozen samples (86). MRD negativity correlates with an improvement in outcome, in terms of PFS and OS (87) in several recent studies, both after first line treatment (69) and in relapsed or refractory MM (88, 89). The analysis of these four phase 3 clinical trials demonstrated that MRD negativity is the most relevant predictor of clinical outcome (90). Moreover, sustained MRD negativity (persistent MRD negativity for 6-12 months) is likely to overcome the poor prognosis of HRMM (91).

For these reasons MRD negativity has represented a primary endpoint for the new randomized clinical trials. Furthermore, the design of a few ongoing clinical trials is MRD-driven, since the duration or the intensification of the treatment is based on the results of MRD.

In the PERSEUS (92) and MASTER (93) trials, a de-escalation of post-autologous maintenance therapy is scheduled; in EMN20 study^5^ including non-transplant-eligible patients, Carfilzomib is discontinued upon reaching MRD negativity. In DRAMMATIC trial^6^ there is a first randomization between maintenance with lenalidomide or Daratumumab plus lenalidomide and a second randomization after two years of treatment, upon evaluating MRD.

Of these clinical studies, only MASTER’s results are mature, underlining the usefulness of an MRD response-adapted consolidation (93). In the nearly future, more data from these studies will allow us to evaluate the usefulness of therapeutic strategies MRD driven.

Some questions are still open: timing of assessment (post induction? post ASCT)?, timing of monitoring and definition of sustained MRD negativity (6 months, 12 months or longer, after MRD negative results)?.

Moreover, MRD in bone marrow does not reflect all the burden of the disease in the body and can be falsely negative in patients with extramedullary disease or with focal bone lesions. For this reason, marrow MRD should be integrated with PET-CT, particularly in those cases with a positive PET-CT before treatment.

New methods of measuring MRD are being studied. Among these, Mass Spectrometry (MS) in the serum, which evaluates the residual monoclonal component, appears to be the most promising, but studies are needed to compare it with standard methods of measuring MRD (94).

At present, in clinical practice, outside trials, MRD is not routinely performed in Italy because only few laboratories have developed the expertise in performing NGS and/or NGF, that are complex and expensive techniques. A network of laboratories able to perform MRD in MM is being created in Italy and the problems linked to the shipping of samples (especially fresh samples required for the execution of NGF, reimbursement of the costs, and coverage of the whole national territory) have not been solved yet.

Rejected recommendation: Minimal residual disease (MRD) results after autologous stem cell transplantation and during maintenance should influence therapeutic decisions.

Round of Consensus: 1

Grade of Consensus: 66.66% (rejected)

Accepted recommendation: At present, Minimal residual disease (MRD) is not yet a tool able to influence therapeutic decisions since it needs to be validated in prospective studies and standardized in clinical practice.

Round of Consensus: 2

Grade of Consensus: 93.33% (accepted)

## Discussion and conclusions

**Figure 4:**
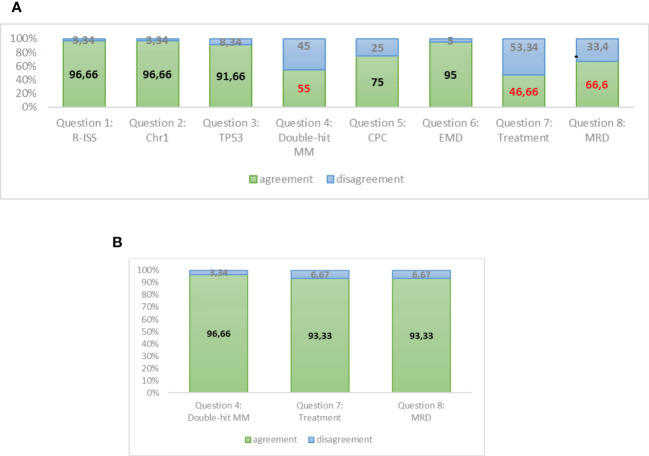
**(A)** Responses to round A of Delphi questionnaire. **(B)** Responses to round B of Delphi questionnaire (assessment reviewed).

Our survey shows (**Figures 4A, B**) a unanimous agreement of the experts on the opportunity of updating the R-ISS staging system in order to better define patients with HRMM.

A strong agreement was reached about the prognostic role of chromosome 1 alteration by FISH and TP53 mutations/deletions by NGS. Moreover, the presence of plasmacytomas at PET-CT and the amount of circulating PC by NGFC should be investigated at diagnosis and should be included in the definition of HR patients. The double-hit category in MM is not well standardized as it is in lymphomas, and it commonly means the association of 2 cytogenetic or molecular alterations, whatever they are. However, the major area of disagreement among the experts is the opportunity to stratify treatment according to the HR. The experts feel that we do not have sufficient data at present to initiate an intensified treatment in HR patients, apart from very rare conditions of PC leukaemias or extramedullary MM that could be treated in a different way in comparison with other MM patients starting from diagnosis. Moreover, at present, clinical data are not considered mature enough to guide treatment decisions during maintenance on the basis of MRD results, since methods need to be standardized and MRD results need to be validated in prospective studies.

In conclusion, the huge progress in the basic and clinical research in MM have made available new prognostic tools, that are well known, accepted, and routinely used by clinicians. However, they are still reluctant to make treatment decisions on the basis of these new prognostic factors and they are waiting for results of prospective trials that integrate HR features and MRD in the decisional algorithm.

## Author contributions

Study concept, design and manuscript: CM, FP, VS, PD. Feedback questionnaire, critical review of the manuscript: AC, DD, VM, MO, MP, RZ. Images: CM, GF. Final approved version of the paper: AC, DD, VM, MO, MP, RZ, FP, CM. Supervised work: FP, RS, RF. Acquisition of the financial support for the project led to this publication: RF, FP, CM. All authors contributed to the article and approved the submitted version.

## Footnotes

1 NIH Cancer Institute (2022); https://seer.cancer.gov/statfacts/html/mulmy.html

2 MAYO Clinic (2022); https://globalmmacademy.com/wp-content/uploads/2021/03/RiskStrat3.0rev_svr_Mayo-clinic.pdf

3
https://clinicaltrials.gov/ct2/show/NCT04483739

4 Clinical Trials (2022); https://clinicaltrials.gov/ct2/show/NCT03896737

5 Clinical Trials (2022); https://clinicaltrials.gov/ct2/show/NCT04096066

6
https://clinicaltrials.gov/ct2/show/NCT04071457
